# Uric Acid Spherulites in the Reflector Layer of Firefly Light Organ

**DOI:** 10.1371/journal.pone.0056406

**Published:** 2013-02-18

**Authors:** King-Siang Goh, Hwo-Shuenn Sheu, Tzu-En Hua, Mei-Hua Kang, Chia-Wei Li

**Affiliations:** 1 Institute of Molecular and Cellular Biology, Department of Life Science, National Tsing-Hua University, Hsinchu, Taiwan; 2 Institute of Biotechnology, Department of Life Science, National Tsing-Hua University, Hsinchu, Taiwan; 3 National Synchrotron Radiation Research Center, Hsinchu, Taiwan; New Mexico State University, United States of America

## Abstract

**Background:**

In firefly light organs, reflector layer is a specialized tissue which is believed to play a key role for increasing the bioluminescence intensity through reflection. However, the nature of this unique tissue remains elusive. In this report, we investigated the role, fine structure and nature of the reflector layer in the light organ of adult *Luciola cerata*.

**Principal Findings:**

Our results indicated that the reflector layer is capable of reflecting bioluminescence, and contains abundant uric acid. Electron microscopy (EM) demonstrated that the cytosol of the reflector layer's cells is filled with densely packed spherical granules, which should be the uric acid granules. These granules are highly regular in size (∼700 nm in diameter), and exhibit a radial internal structure. X-ray diffraction (XRD) analyses revealed that an intense single peak pattern with a d-spacing value of 0.320 nm is specifically detected in the light organ, and is highly similar to the diffraction peak pattern and d-spacing value of needle-formed crystals of monosodium urate monohydrate. However, the molar ratio evaluation of uric acid to various cations (K^+^, Na^+^, Ca^2+^ and Mg^2+^) in the light organ deduced that only a few uric acid molecules were in the form of urate salts. Thus, non-salt uric acid should be the source of the diffraction signal detected in the light organ.

**Conclusions:**

In the light organ, the intense single peak diffraction signal might come from a unique needle-like uric acid form, which is different from other known structures of non-salt uric acid form. The finding of a radial structure in the granules of reflector layer implies that the spherical uric acid granules might be formed by the radial arrangement of needle-formed packing matter.

## Introduction

Uric acid is a waste product from the metabolism of nucleotides [1]–[3]. In human and other mammals, uric acid can be further metabolized into final waste products, urea and glyoxylic acid by enzymes, such as uricase [3]. However, elevation of uric acid concentration in blood might result in gout and kidney stone [4]. Gout is a type of arthritis caused by the accumulation of needle-like crystals of urate salt–monosodium urate monohydrate–in the joints [5]. Various artificial uric acid or urate crystals have been extensively studied [6]–[9]. However, the physical property and formation of uric acid or urate crystals in the biological system, except monosodium urate monohydrate [10]–[12], are still poorly understood.

Unlike mammals, birds, insects and reptiles can directly excrete uric acid out of their body [1], [2], [13]. In 1969, the morphology of excreted uric acid in bird's dropping was first described [14]. The uric acid excreted by birds is a group of spherical particles with a various sizes ranging from 0.5 to 10 µm [14], [15]. Following studies demonstrated that these tiny particles exhibit a unique single peak X-ray diffraction (XRD) pattern, and are believed to be a liquid-crystal like matter [16], [17].

For insects, uric acid is more than a waste product. Uric acid stored in specialized urate cells within insect's fat body can be utilized as a nitrogen source for synthesizing amino acids and nucleotides [18]. Interestingly, in some insects, uric acid can also be served as a white pigment. For instance, larvae of armyworm, *Pseudaletia separata,* use uric acid stored in epidermal cells to form white stripes on their body [19]. Larvae of silkworm, *Bombyx mori*, accumulate uric acid in hypodermal cells to form a gray opaque skin [20]. For some butterfly species, uric acid derivatives are used as a white dye on their wings [21].

Adult fireflies (Coleoptera: Lampyridae) have a specialized abdominal light organ to produce bioluminescent mating signal [22], [23]. The light organ is a slab-like tissue composed of a ventral photogenic layer and a dorsal reflector layer [24]–[28]. Photogenic layer is the source where the bioluminescence occurs [25]–[28]. Reflector layer is thought to be a specialized tissue for increasing bioluminescence intensity via reflection; this layer is formed by a group of cells filled with opaque white granules [25]–[28]. The nature of these opaque granules is thought to be urate, but it has only been studied a century ago [27]. In this report, we utilized modern techniques such as biochemical assay, electron microscopy and XRD to examine the nature and the morphology of the reflector layer of adult *Luciola cerata*.

## Results

### Weak Bioluminescence Emitting from Dorsal Light Organ

**Figure 1:**
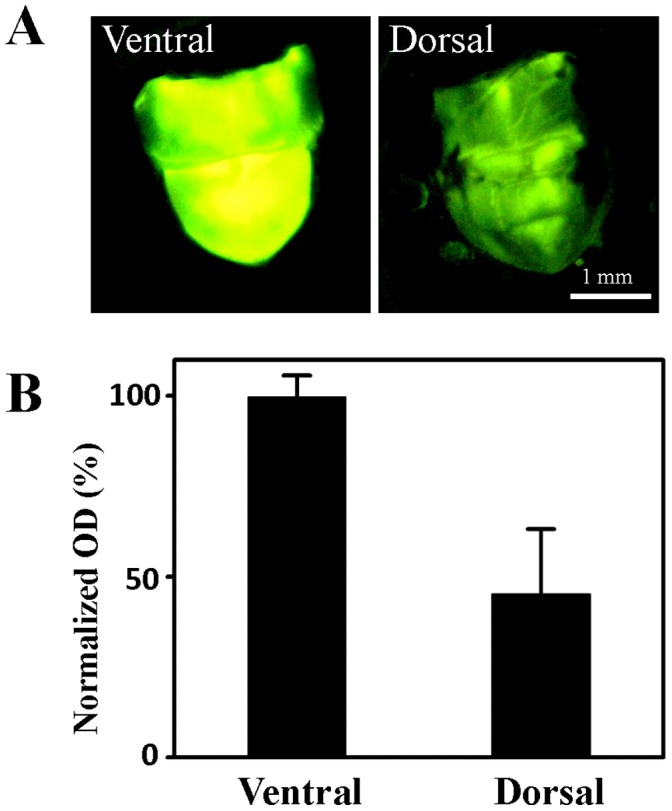
Light Intensity measurement of dorsal and ventral light organs. A) Ventral view (left panel) and dorsal view (right panel) of an isolated luminescent light organ. The light organ (including photogenic layer and reflector layer) was dissected from the 6^th^ and 7^th^ body segments of a dying *L. cerata*. B) Evaluation of light intensity from the ventral and dorsal luminescent light organs. The averaged light intensity (optical density, O.D) of ventral or dorsal light organs was evaluated from at least three images using ImageJ, and normalized by the averaged light intensity of ventral light organ.

*In vivo* study of the function of the reflector layer in an adult firefly is difficult due to the limitation of bioluminescence control. However, we found that the light organs isolated from dying fireflies could be used for the functional studying of the reflector layer. In recording adult firefly life-span, we noticed that a few dying *Luciola cerata* (about 3 in 100 individuals) generated flashes into a continuous glowing, which could stably sustain at least 2 hours. The luminescent light organs of these fireflies were successfully isolated, and utilized for the evaluation of the light intensity from ventral (left panel in Fig. 1A) and dorsal (right panel in Fig. 1A) light organ. It was estimated that the light intensity from dorsal light organ is approximately 45% of that from the ventral light organ (Fig. 1B). This result indicates that the reflector layer located at the dorsal light organ should be able to effectively reflect the light emitting from the ventral photogenic layer.

### Specific Abundance of Uric Acid in the Reflector Layer of Light Organ

**Figure 2:**
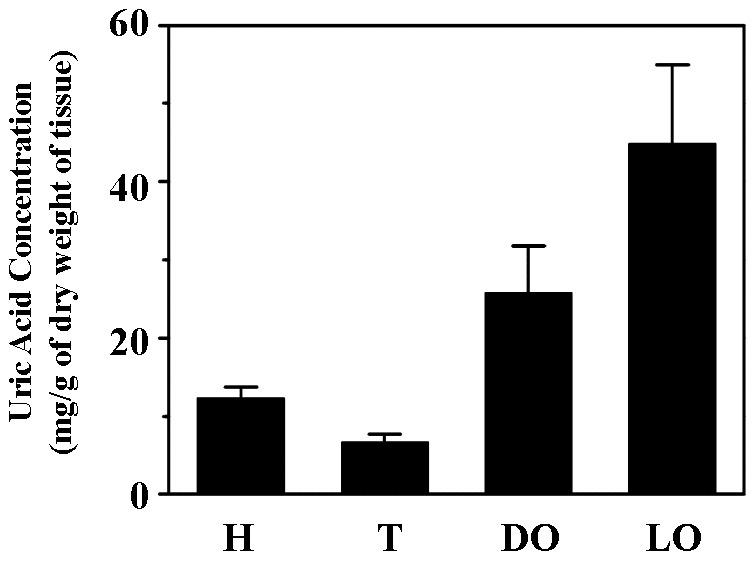
Uric acid concentration evaluation of the light organ and other firefly tissues. Dry weight of each isolated firefly body parts, including head (H), thorax (T), dorsal organ (DO) and light organ (LO) were measured. Each dried body part was homogenized to evaluate the uric acid concentration (mg/g of dry weight of tissue) using the uric acid assay kit. Shown are the mean and standard derivation for three samples.

The nature of the reflector layer has only been investigated by less-specific chemical tests a century ago and was presumed to be urate [27]. To validate the presumption, we used a commercial enzymatic (uricase) assay kit that could specifically detect uric acid or urate. The light organ and other firefly body parts, including dorsal organ, thorax and head of *L. cerata*, were dissected as we reported previously [24], and further prepared for the evaluation of uric acid or urate concentration. It was estimated that the concentration (mg/g of dry weight of tissue) of uric acid or urate is about 44.80 in the light organ, 25.85 in the dorsal organ, 6.74 in the thorax, and 12.32 in the head (Fig. 2). This result clearly shows that the light organ is the tissue that contains the most abundant uric acid or urate.

**Figure 3:**
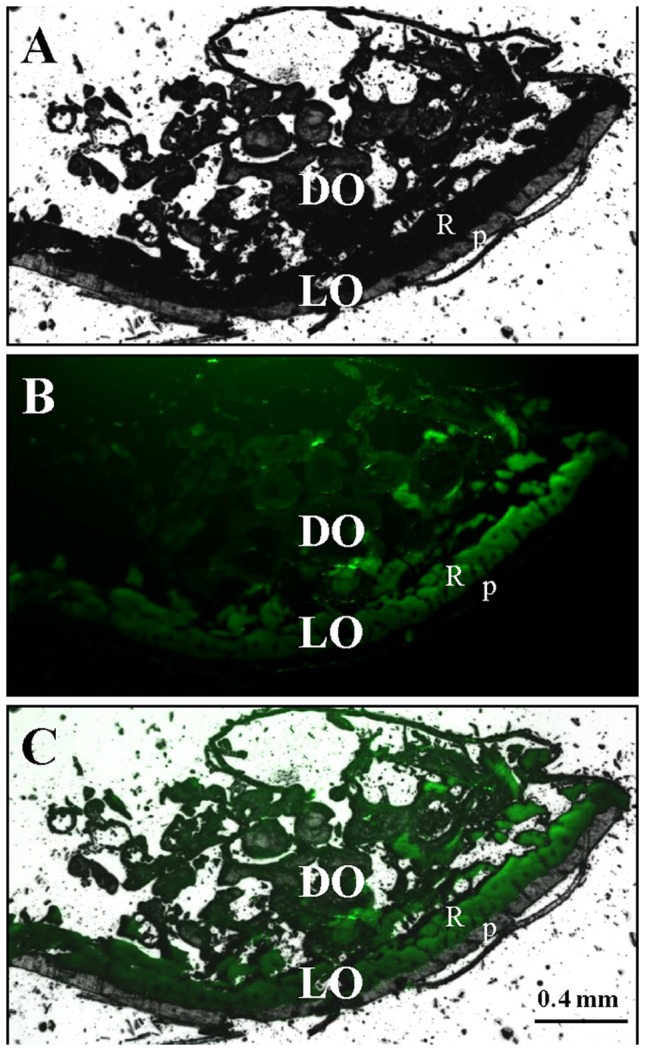
Tissue-specific localization of uric acid in the light organ. A) DIC micrograph of a cross-section from the 6^th^ body segment of *L. cerata*. Light organ (LO), localized at the abdominal tissue section, consists of a translucent photogenic layer (P) and an opaque reflector layer (R). The dorsal organ (DO) localized at the dorsal light organ possesses various tissues. B) Fluorescent micrograph of the same tissue section treated by uric acid assay kit. The uric acid signal is specifically intense in the reflector layer (R), but none in the photogenic layer (P). C) Merged micrograph of A and B.

We further investigated the tissue-specific localization of uric acid or urate in the light organ using fluorescence microscopy. The presence of uric acid/urate was detected by uric acid/urate dependent uricase catalyzed chemical luminescence. DIC micrograph revealed that abdominal light organ is composed of an opaque reflector layer and a translucent photogenic layer (Fig. 3A). Fluorescent micrograph showed intense uric acid or urate signal in the reflector layer, and weak in the dorsal organ, but none in the photogenic layer (Fig. 3B and C). These results confirm that uric acid or urate is specifically abundant in the reflector layer.

### Spherical Granules of Consistent Diameter in the Reflector Layer

**Figure 4:**
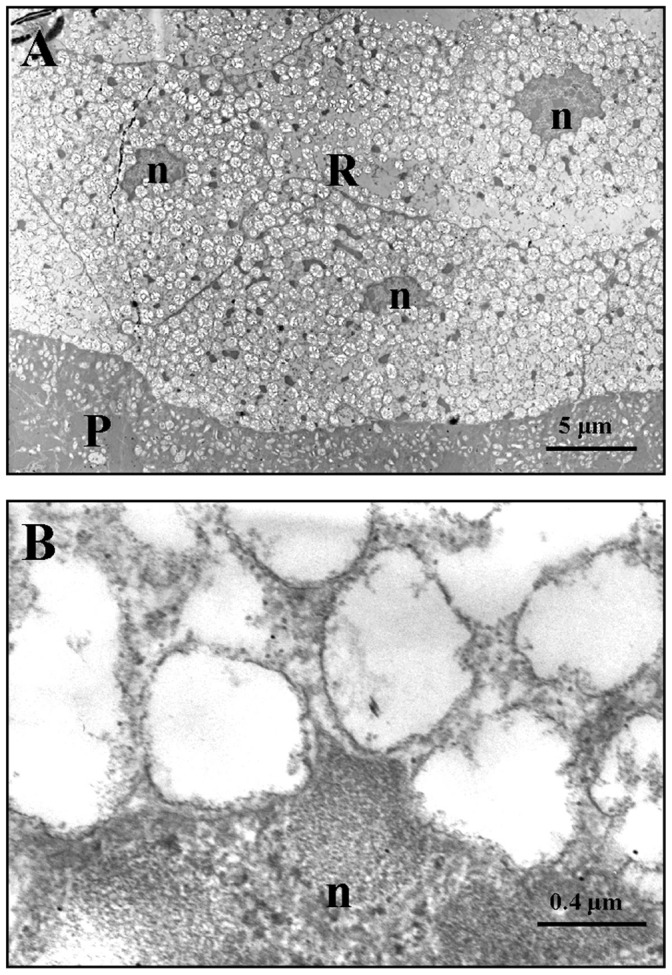
TEM micrographs of the reflector layer in the light organ. A) Micrograph of a bordering region between photogenic layer (P) and reflector layer (R) in the light organ of *L. cerata*. Densely packed granules are found specifically in the reflector layer, but none in the photogenic layer. B) Micrograph of a bordering region between the cytosol containing granules and the cell nuclei (n) in the reflector layer.

Fine structure of the reflector layer of *L. cerata* was studied using both transmission electron microscopy (TEM) and scanning electron microscopy (SEM). TEM showed that the reflector layer consists of cells with an average size of about 40 µm in length and nearly 15 µm in thickness (Fig. 4A). The cytosol of these cells is densely packed with numerous spherical granules (Fig. 4A). All these intracellular granules show an empty internal space, and are surrounded by a layer of membrane-like structure (Fig. 4B). These granules should be the site for uric acid or urate storage as previous descriptions [25]–[28].

**Figure 5:**
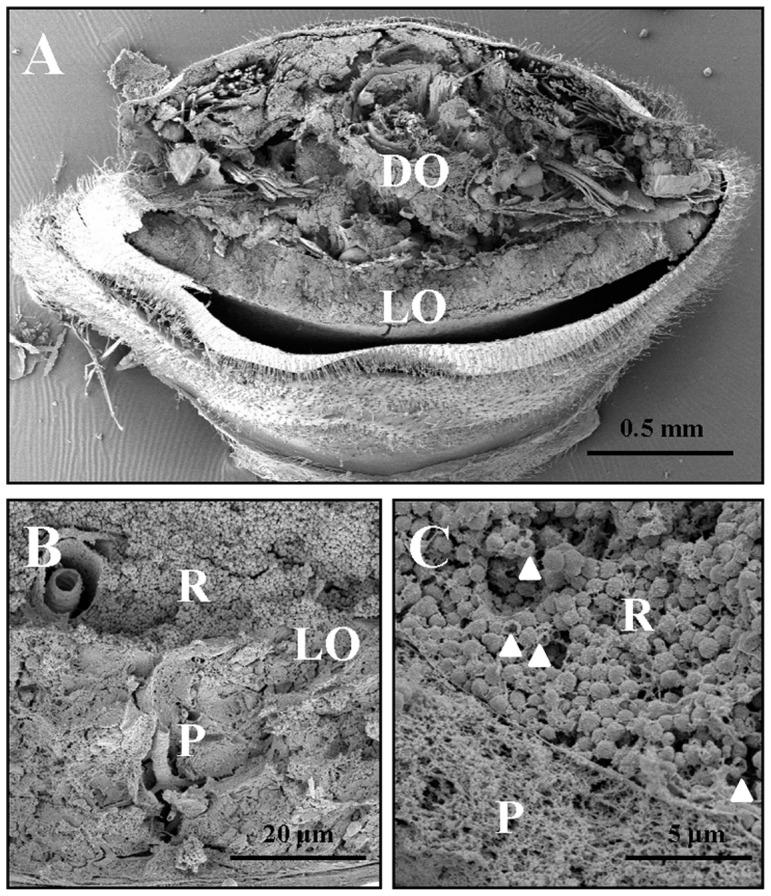
SEM micrographs of the reflector layer and the photogenic layer in the light organ. A) Light organ (LO), located at the abdominal tissue section (the 6^th^ body segment), is a slab-like tissue with a thickness about 240 µm. B) The photogenic layer (P) located at the ventral light organ is a 40 µm thick tissue, and shows a morphology distinct from that of the reflector layer (R). C) In the reflector layer (R), densely packed spherical granules are found, and some of them had become hollowed (indicated as arrows). DO: Dorsal organ.

**Figure 6:**
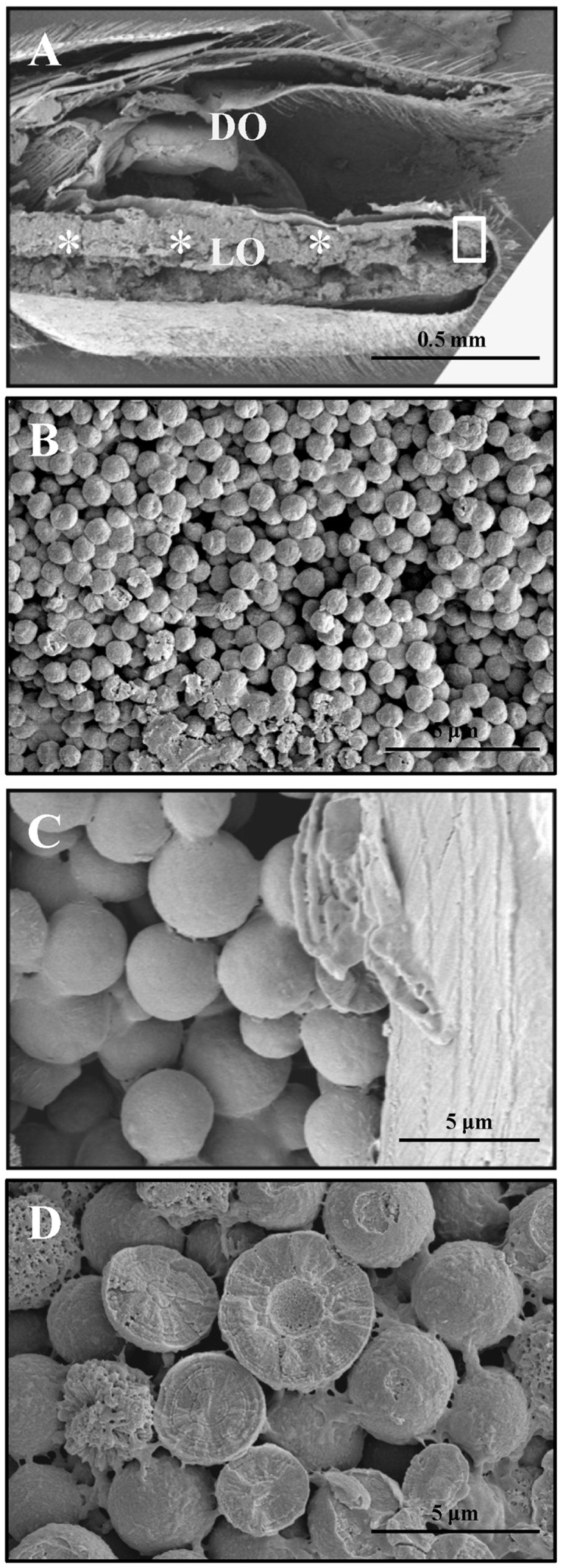
Size screening of spherical granules in the reflector layer of light organ by SEM. A) The sizes of spherical granules in the reflector layer were investigated in the distinct regions (marked with * and square) of a transversal tissue section prepared from the 7^th^ segment of *L. cerata*. B) The granules in the * region are highly regular in size, about 700 nm in diameter. C) The granules at the edged region of reflector layer (square region) are unusually large with varying sizes. D) The fractures of unusual large granules.

A rough measurement by SEM showed that the light organ consists of a 200 µm thick reflector layer, and a 40 µm thick photogenic layer (Fig. 5A and B). Photogenic layer and reflector layer can be distinguished by their distinct morphology (Fig. 5B and C). The photogenic layer reveals a net-like internal structure, and the reflector layer contains numerous spherical granules, which should be the uric acid or urate granules. In the reflector layer, we also found that some granules had become hollowed, which revealed a vesicle-like structure (indicated by arrows in Fig. 5C). Through a rough screening (Fig. 6A), we discovered that most of the granules in the reflector layer are highly regular in size, about 700 nm in diameter (Fig. 6B). However, some unusual large granules, whose diameter could be up to 4 µm, were found specifically in the edge of the reflector layer (indicated by square in Fig. 6C). The fractures of these large granules showed a radial internal structure consists of multiple concentric layers (Fig. 6D).

### Specifically Detection of an Intense Single-peak XRD Signal in the Light Organ

**Figure 7:**
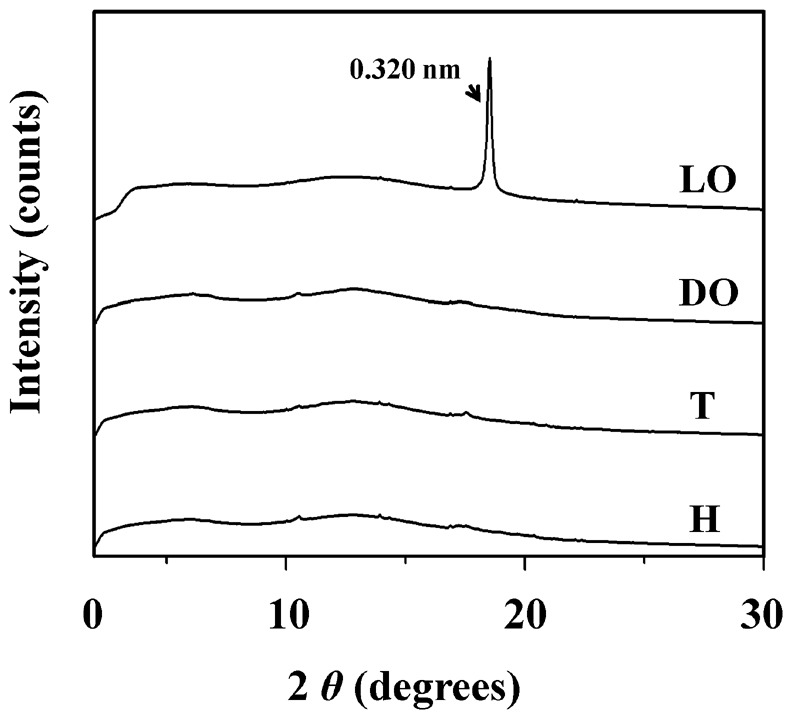
The X-ray diffraction (XRD) patterns of the light organ and other tissues of *L. cerata*. Homogenized tissues of the light organ (LO), the dorsal organ (DO), the thorax (T) and the head (H) were prepared for XRD analyses. The d-spacing value of 0. 320 nm, corresponding to the diffraction peak of light organ, is indicated.

To investigate if uric acid or urate stored in the light organ of *L. cerata* is crystallized, homogenized light organ and other firefly body parts were prepared for X-ray diffraction (XRD) analyses. An intense diffraction signal was detected only in light organ, but none in dorsal organ, thorax and head (Fig. 7). The detected diffraction signal reveals a single-peak pattern. The d-spacing value corresponding to the intense peak of the diffraction signal was determined to be 0.320 nm. This diffraction signal is identical in the peak pattern and the d-value to those of spherical uric acid particles found in the bird dropping [16], [17], indicating that the spherical uric acid granules stored in the reflector layer should be the source of diffraction signal. In addition, the re-crystallized matters of the reflector layer exhibit a plate-like transparent morphology (Fig. S1A), and were also examined by XRD technique. The result revealed that the diffraction signal of the re-crystallized matters is identical to that of uric acid dihydrate crystals (data not shown).

**Figure 8:**
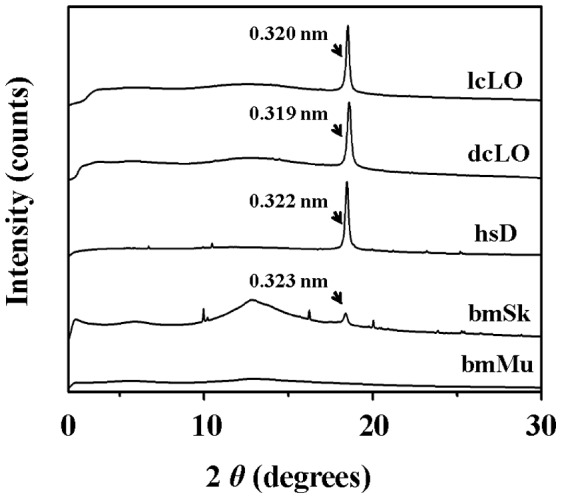
The X-ray diffraction (XRD) patterns of firefly light organs and other animal tissue and dropping containing uric acid. The samples, including homogenized light organs from fireflies, *L. cerata* (lcLO) and *Diaphanes citrinus* (dcLO), homogenized skin from silkworm larvae, *Bombyx mori* (bmSK), and the white matters from the dropping of gecko, *Hemidactylus stejnegeri* (hsD), were prepared for XRD analyses. The d-spacing values of the analyzed tissues and dropping, which are similar in the d-spacing value and the peak position to those of the light organ of *L. cerata* (LO), are indicated, respectively. Homogenized muscle of silkworm larvae, *B. mori*, (bmMU), is served as a reference tissue.

We further explored if the single-peak diffraction signal detected in the light organ of *L. cerata* could be also found in other reported biological sources that contain uric acid or urate. Previous studies indicated that the silkworm larvae skin and the reptile dropping also contain spherical granules or particles of uric acid or urate [20], [29]. Hence, homogenized light organs from distinct firefly species, *L. cerata* and *Diaphanes citrin,* dissected skin from silkworm larvae, *Bombyx mori*, and the white matter (excreted uric acid) from the dropping of gecko, *Hemidactylus stejnegeri,* were prepared for XRD analyses. The result showed that the single-peak diffraction signal is intense in firefly light organs and in the dropping of gecko (Fig. 8). In the silkworm larvae skin, a weak diffraction signal identical in peak position to that of firefly light organs was also detected (Fig. 8). The d-spacing values for these tissues and the dropping were determined into a range from 0.319 nm to 0.323 nm. The muscle tissue of *B. mori* was used as a reference tissue that lacks of uric acid or urate, and no diffraction signal was detected (Fig. 8). These results clearly shows that the unique single peak diffraction signal is detected not only in firefly light organs but also in other animal tissue or dropping containing spherical granules or particles of uric acid or urate.

### Detection of Highly Similar XRD Signals in Both Light Organ and Monosodium Urate Monohydrate

**Figure 9:**
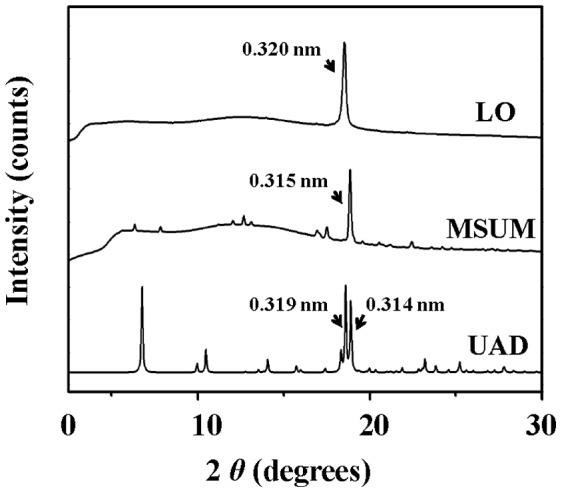
The XRD patterns of the light organ and other artificial uric acid or urate crystals. Needle-formed crystals of monosodium urate monohydrate (MSUM) and plate-formed crystals of uric acid dihydrate (UAD) were prepared as described in Method and Material. The d-spacing values for the intense peak of MSUM and UAD, similar to that of the light organ of *L. cerata* (LO), are indicated, respectively.

We further investigated the similarity in XRD signal between the material in the light organ of *L. cerata* and other known structures of uric acid or urate crystals. Needle-formed crystals (Fig. S1B) of monosodium urate monohydrate (MSUM) and plate-formed crystals (Fig. S1C) of uric acid dihydrate (UAD) were prepared as previous reports [6], [30]. The diffraction peak pattern of light organ is almost identical to the strongest peak of MSUM but has a little shift to lower angle compared to MSUM (Fig. 9). In addition, this single peak pattern is apparently distinct from the multiple-peak pattern of UAD (Fig. 9). The d-spacing value (0.320 nm) for the light organ is slightly distinct from that (0.315 nm) for MSUM and the two major peaks (0.319 and 0.314) for UAD (Fig. 9). The highly similar in the peak pattern indicates that both the structures of the material in the light organ and MSUM might be similar.

In addition, we also investigated if the material in the light organ has a phase change when it is heated by using differential scanning calorimetry (DSC). The result showed that no endo- or exothermic peak signal was found in the heating process (from 25°C to 300°C) and cooling process (from 300°C to 25°C) for the light organ (Fig. S2A) and the excreted uric acid from the dropping of gecko (Fig. S2B), indicating that both the material in the light organ and the excreted uric acid of gecko have no phase change and are stable under heating and cooling process.

To examine if the material in the light organ is in the form of urate salt, we determined the concentration of various cations (K^+^, Na^+^, Ca^2+^ and Mg^2+^) in the light organ of *L. cerata* using inductively coupled plasma mass spectrometry (ICP-MS), and further evaluated the molar ratio of uric acid to each cation in the light organ (Table 1). It was estimated that the concentration (mg/g of dry weight of tissue) of K^+^ is 3.01×10^−3^, that of Na^+^ is 0.84×10^−3^, that of Ca^2+^ is 0.19×10^−3^, and that of Mg^2+^ is 0.16×10^−3^. Based on the determined concentration of uric acid in the light organ (Fig. 2), we evaluated the molar ratio of uric acid to these cations in the light organ. The molar ratio of uric acid to K^+^ is about 3454∶1, to Na^+^ is about 7297∶1, to Ca^2+^ is about 40583∶1, and to Mg^2+^ is about 57750∶1. The high molar ratio of uric acid to each cation indicated that only very few uric acid molecules in the light organ could form urate salt with cations. Thus, the materials in the light organ should not be in the form of urate salt but probably non-salt uric acid.

**Table 1:** Molar ratio of uric acid to various cations in the light organ.

Component Name (mass)	Concentration (mg/g of dry weight of tissue)	Molar Ratio (uric acid : cation)
Uric acid (168)	44.799	–
K^+^ (39)	3.010×10^−3^	3454∶1
Na^+^ (23)	0.840×10^−3^	7297∶1
Ca^2+^ (40)	0.185×10^−3^	40583∶1
Mg^2+^ (24)	0.158×10^−3^	57750∶1

## Discussion

Although the light organ of adult fireflies has been extensively studied over a century, the nature of reflector layer remains obscure [26]. In the present study, we demonstrate that the reflector layer is capable of reflecting light, and confirm that this unique tissue layer is made of tiny intracellular granules containing uric acid.

The role of reflector layer could be investigated because we discovered that several *Luciola cerata* individuals converted flashes into a continuous glow when they were dying. Similar phenomenon has never been reported before, and the cause of this phenomenon remains unclear. We successfully isolated the luminescent light organs from those dying fireflies, and further used them for *in vivo* functional study of the reflector layer. Our result indicates that the light intensity from the dorsal light organ is relatively weaker than that from the ventral light organ (Fig. 1A and B). This result suggests the reflector layer located at the dorsal photogenic layer should be a functional light reflector as previously proposed [27], [28].

We demonstrated the reflector layer is the tissue containing the most uric acid in a *L. cerata* (Fig. 2 and 3). Uric acid stored in the firefly reflector layer is a white opaque matter under white light illumination (data not shown), and thus, the stored uric acid should be able to reflect visible light (spectrum from 400 to 750 nm), including firefly bioluminescence (spectrum from 540 to 580 nm) [31]. Other insects utilize uric acid as a white pigment to form white stripes or skin color [19], [20], suggesting that uric acid should be an excellent light reflecting material for insects.

Both TEM and SEM studies demonstrated that the cells in the reflector layer of *L. cerata* contains densely packed spherical granules in their cytosol (Fig. 4 and Fig. 5). All these granules exhibit an empty internal space (Fig. 4), which are morphologically similar to that of cytosolic uric acid granules reported in other insect tissues [19], [20]. Thus, we deduce that the empty internal space of the granules in the reflector layer should be the place where uric acid is stored. In the reflector layer, most uric acid granules are highly similar in size, about 700 nm in diameter (Fig. 6A and B). In addition, it was estimated that the reflector layer is about five times thicker than the photogenic layer (Fig. 5A and 5B). We believe that the dense packing of tiny uric acid granules of similar size in a thick reflector layer allows the tissue to become a physical barrier for bioluminescence. We also found some unusually large granules with varying sizes (Fig. 6C and D) localized at the edge of the reflector layer (indicated by square in Fig. 6A), but they do not seem to affect the role of reflector layer.

In the cells of reflector layer, spherical uric acid granules might be coated with a lipid membrane, according to the following findings. First, the TEM study demonstrated that the granules were surrounded by a membrane-like layer (Fig. 4B), which might be a lipid bilayer. Second, some hollowed granules were found (Fig. 5C), and their membrane-like remnant might be vesicles. Thus, the spherical uric acid granules might be originated from organelle. Since degradation of uric acid occurs in peroxisomes [3], [32], it is possible that the cells of reflector layer in differentiating stage suspend the metabolism of uric acid, causing the deposition of uric acid in peroxisomes. Finally, peroxisomes that accumulate large amount of uric acid become opaque granules. However, this hypothesis remains further investigation.

Among various tissues of *L. cerata,* we demonstrated that the light organ is the only one exhibiting the intense diffraction signal, a single-peak pattern with a d-spacing value of 0.320 nm (Fig. 7). Moreover, we found that this single peak diffraction signal was detected not only in the light organs of fireflies, *L. cerata* and *D. citrinus*, but also in other animal tissue and dropping containing spherical granules or particles of uric acid (Fig. 8). All single peak diffraction signals detected in this study are almost identical to that reported in the study of spherical uric acid particles in the bird dropping [16], [17]. This implied that the nature and formation mechanism of these spherical uric acid granules or particles in fireflies and other animals might be evolutionary conserved.

Uric acid or urate salt is a flat molecule [2], [6]. Thus, the single-peak diffraction signal detected in the light organ might come from a short face-face stacking axis formed by uric acid molecules. The types of face-face interaction are found in all known structures of uric acid and urate salt [6], [33], but it doesn't mean that the material in the light organ corresponds to any of the known structures. We demonstrated both the light organ and the needle-formed monosodium urate monohydrate (MSUM) exhibited a highly similar single peak diffraction pattern (Fig. 9), suggesting that the material in the light organ might be a needle-formed matter. However, the molar ratio evaluation of uric acid to various cations (K^+^, Na^+^, Mg^2+^ and Ca^2+^) in the light organ indicated that the amount of cations in light organ is not enough for the formation of urate salt (Table 1). Therefore, the material in the light organ is apparently not MSUM or other urate salts but probably non-salt uric acid. However, the single peak diffraction pattern of the light organ is also distinct from those multiple-peak patterns of plate-formed crystals of uric acid dihydrate (Fig. 9) and other reported non-salt uric acid crystals [6]. This result clearly shows that the material in the light organ is a unique uric acid form different from those known structures of non-salt uric acid crystals. In addition, the single peak diffraction pattern also implies that the uric acid form in the light organ is lack of three dimensional order. Similar single peak diffraction patterns were usually detected in the liquid-crystal formed matters [34]–[36], but this doesn’t represent that the uric acid form in the light organ is also a liquid-crystal. Through the DSC analysis for the material in the light organ (Fig. S2A), we didn't detect any endo- or exothermic signal in the heating or cooling process, suggesting that the uric acid form in the light organ is a stable matter under heating and shouldn't be a liquid-crystal.

In the SEM study, we showed that the fractures of uric acid granules in the reflector layer reveal a radial internal structure (Fig. 6D). Similar internal structures were also found in the uric acid particles from the bird dropping [15], and in the uric acid granules stored in the body wall of sea squirt [37]. This indicates that the granules in reflector layer might be constituted by a radial arrangement of needle-formed uric acid. This morphology character (radial internal structure) is consistent with that of those spherulites [38], [39]. Spherulites are a group of spherical solids that ehibit densely branched, polycrystalline solidification internal patterns, and ubiquitously found in nature, such as simple organic liquids, liquid crystals and diverse biological molecules [39]. Hence, the uric acid form in the granules of the reflector layer might be in spherulite phase.

Based on the results in this study, we hereby propose a structural model for the uric acid granules in the reflector layer–the spherical granule, which is encapsulated by a lipid bilayer, is constituted by a radial arrangement of needle-formed uric acid (or a uric acid spherulite).

### Conclusions

We showed that firefly reflector layer is a functional tissue for increasing light intensity, and specifically contains abundant uric acid. The specific detection of intense single peak diffraction signal in the light organ might come from a unique needle-like uric acid form, which is different from other known structures of non-salt uric acid form. The finding of a radial structure in the granules of reflector layer suggests that the spherical uric acid granules might be constituted by the radial arrangement of needle-formed packing matter or the uric acid spherulite.

## Materials and Methods

### Chemical

All chemicals were purchased from Sigma-Aldrich (MO, USA) unless indicated otherwise.

### Animals

For this study, both male adult fireflies, *Luciola cerata* and *Diaphanes citrinus,* were collected from the unlit area (24°37'53.56"N 121°61'35.76"S) of Nanjhuang township of Miaoli county (Taiwan) after sunset from April to September. No specific permit was required for the described field studies, because both firefly species, *L. cerata and D. citrinus*, do not belong to endangered or protected species (http://conservation.forest.gov.tw/mp.asp?mp=11), and the sampling place is not belonged to private owner or natural preservation zones (http://conservation.forest.gov.tw/lp.asp?ctNode=725&CtUnit=601&BaseDSD=7&mp=11). Silkworm larvae, *Bombyx mori*, were a gift from Dr. Chun-Jung Chen. The protocol used in this study had been consulted with National Tsing-Hua University laboratory animal room and was in strict accordance with the R.O.C animal protection act (Act number: 10000136211, http://law.coa.gov.tw/GLRSnewsout/EngLawContent.aspx?Type=E&id=120). Specimens, except those fireflies for the recording life-span, were sacrificed with CO_2_ asphyxiation and stored at −80°C before used.

### Uric Acid Concentration Measurement

To evaluate the uric acid amount in firefly tissues, the *L. cerata* was dissected into four body parts including the light organ (both 6^th^ and 7^th^ segments), the head, the thorax, and the dorsal organ as we reported previously [24]. All dissected tissues were dried in 60°C in vacuum overnight and followed by a precisely weighing. The tissues were then homogenized in a 250 µl solution containing 50 mM sodium phosphate, pH 8.0. After heating at 100°C for 10 min and then centrifugated in 12000 rpm for 10 min at room temperature (RT), the supernatant was collected for uric acid concentration assay. The uric acid concentration of firefly tissues was determined using an enzymatic assay kit (Uric acid assay kit, BioVision, CA) containing uricase and a fluorescent probe. In this assay, uricase catalyzes the oxidation of uric acid, which produces H_2_O_2_ that could activate the probe to emit the fluorescence. Based on this principle, uric acid can be detected or evaluated. All procedures were followed the operating manual. Uric acid level was measured using a fluorometric (Perkin Elmer Wallac Victor 1420 multilabel counter, MA) to detect the intensity of signal at Ex/Em  = 535/587 nm.

### Fluorescence Microscopy

The 6^th^ body segment of *L. cerata* was isolated and fixed in a fixation solution containing 140 mM NaCl, 50 mM sodium phosphate, and 4% paraformaldehyde at pH 7.2 for 4 hr at RT, and then washes with PBS three times for 5 min each. The specimens were then subjected to serial ethanol and acetone dehydration. The ethanol concentrations were from 30%, 45%, 60%, 75%, 90%, to 100%, and followed acetone from 30%, 60%, 90% to 100% for 25 min each step at RT. Acetone-dehydrated specimens were then infiltrated by xylene with gradually increased xylene ratio from 25%, 50%, 75% to 100%. The xylene-treated specimens were transferred into a pre-heated embedding wax (Paraplast, Electron Microscopy Sciences, PA), and incubated at 60°C for 15 hr. The tissue-embedded wax was sectioned with a microtome (Microm HM 320, Germany) into 20 µm thickness sections. The sections were de-waxed by immersing the samples in xylene solution, and stored in a dry box at RT before used. For uric acid detection, the detecting solution containing uricase and fluorescence probe was freshly prepared from Uric acid assay kit (BioVision, CA). After immersed the tissue sections in the detecting solution for 1 min at RT, the images were obtained using a fluorescence microscopy (Zeiss Observer Z1, Germany) to detect the fluorescent signal at Ex/Em  = 535/587 nm.

### Sample Preparation for Electron microscopy

The isolated light organs of *L. cerata* were primary fixed in a fixation solution containing 140 mM NaCl, 50 mM sodium phosphate, 2.5% glutaraldehyde and 4% paraformaldehyde, pH 7.2, for 4 hr at RT. After washing with PBS three times for 5 min each, the specimens were subsequently immersed in 1% osmium tetraoxide (OsO_4_) for secondary fixation, and then washed with PBS three times for 5 min each. Serial dehydration was carried out with ethanol concentration increased from 30%, 45%, 60%, 75%, 90%, to 100% for 25 min each step at RT. Dehydrated specimens were washed by 100% ethanol two times for 25 min each at RT. The specimens were then used for the electron microscopy.

### Scanning Electron microscopy (SEM)

After remove the excessive ethanol, the dehydrated specimens were pre-dried in vacuum at 60°C overnight. The specimens were mounted on an aluminum stub with double stick tape, and were further dried by critical point-drier before sputter coated with gold by sputter coater (Nanotech SEMPREP 2, IL). The image obtained using a scanning electron microscopy (Hitachi S4700, Japan) interfaced with an image-analyzing computer.

### Transmission Electron Microscopy (TEM)

The ethanol immersed specimens were embedded with Spurr’s low viscosity resin (Electron Microscopy Sciences, PA) after the dehydration. The curing condition was 60°C for 16 hr. Ultrathin sections with 90 nm thickness were obtained using ultra-microtome (Leica Ultracut R, Germany). TEM images were obtained using a transmission electron microscope (Hitachi H-7500, Japan) after double stained with uranyl acetate and lead citrate.

### Sample Preparation for XRD and DSC Analyses

The light organs of fireflies, *L. cerata, and D. citrinus*, the skin and muscle of silkworm larva, *B. mori*, were dissected and homogenized for direct analyses. Excreted uric acid (white matters) in the dropping of gecko, *Hemidactylus stejnegeri,* were collected from the residence, and stored in −20°C before used. Plate-formed crystals of uric acid dihydrate and needle-formed crystals of monosodium urate monohydrate were prepared and stored in the condition described previously [6], [30]. To prepare re-crystallized matters of firefly light organs, the solution containing 0.5 ml ddH_2_O and 1 mg (wet weight) homogenized light organs was incubation at 60°C with vigorous shaking for 15 min for dissolving uric acid. After centrifugated in 12000 rpm at room temperature for 15 min, supernatants were collected. The re-crystallization was carried out by evaporating the collected supernatant at RT until the precipitation appeared. The precipitated crystals were collected after sieving, and stored in the condition described previously [6] before used.

### X-ray Diffraction (XRD) and Differential Scanning Calorimetry (DSC)

X-ray diffraction was performed at the BL01C2 beamline of National Synchrotron Radiation Research Center (NSRRC, Taiwan) as previously reported [40]. The d-spacing value (lattice distance) of the crystals was obtained from the *Bragg* condition is nλ  = 2dsin(θ). The wavelength λ is 0.9918 Å (12.5 keV), n is the order of diffraction (n = 1), and θ is the *Bragg* angle. Differential scanning calorimetric studies were carried out on a power compensation Perkin Elmer DSC-7 (Norwalk, CT, USA). The samples were analyzed under the heating or cooling at the rate of 5°C/min.

### Inductively Coupled Plasma-mass Spectrometry (ICP-MS)

The dissected light organs of *L. cerata* were dried at 60°C in vacuum overnight. The dried specimens were homogenized and then weighed precisely. The sample was digested with concentrated nitric acid (HNO3) in a microwave oven (Mars-5X, CEM, USA). The concentrations of metals (Na^+^, K^+^, Mg^2+^ and Ca^2+^) were analyzed using an ICP-MS (Agilent 7500ce, Japan). Standard solutions (E. Merck, Darmstadt, Germany) were used to determine elemental concentrations.

## Supporting Information

Figure S1 **Dark field microscopic images of crystalline matters.** A) Re-crystalline matters of light organ of *L. cerata*. B) Needle-formed crystals of monosodium urate monohydrate. C) Plate-formed crystals of uric acid dihydrate.
(TIF)

Figure S2 **DSC thermographs of firefly light organ and the excreted uric acid of gecko.** A) The light organ of *L. cerata* and B) the excreted uric acid of *H. stejnegeri* are examined under a heating (black line) and cooling (gray line) process.
(TIF)
