# Supplementary figures and images for: Uric Acid Spherulites in the Reflector Layer of Firefly Light Organ
Source: PLoS One. 2013 Feb 18;8(2):e56406. doi: 10.1371/journal.pone.0056406 (PMC3575340; doi:10.1371/journal.pone.0056406)

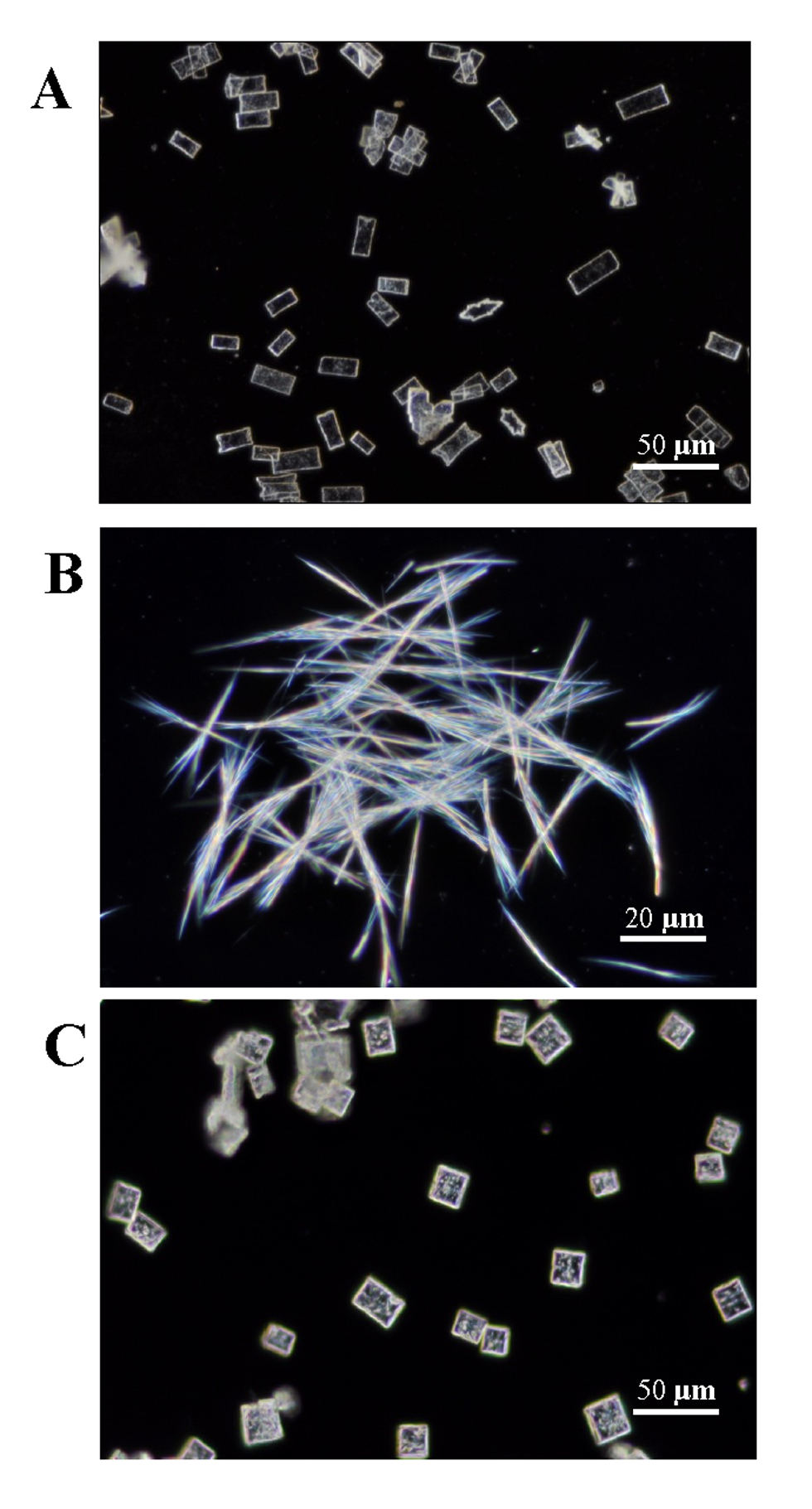

Supplement: Figure S1 — Dark field microscopic images of crystalline matters. A) Re-crystalline matters of light organ of L. cerata. B) Needle-formed crystals of monosodium urate monohydrate. C) Plate-formed crystals of uric acid dihydrate. (TIF) [file pone.0056406.s001.tif]

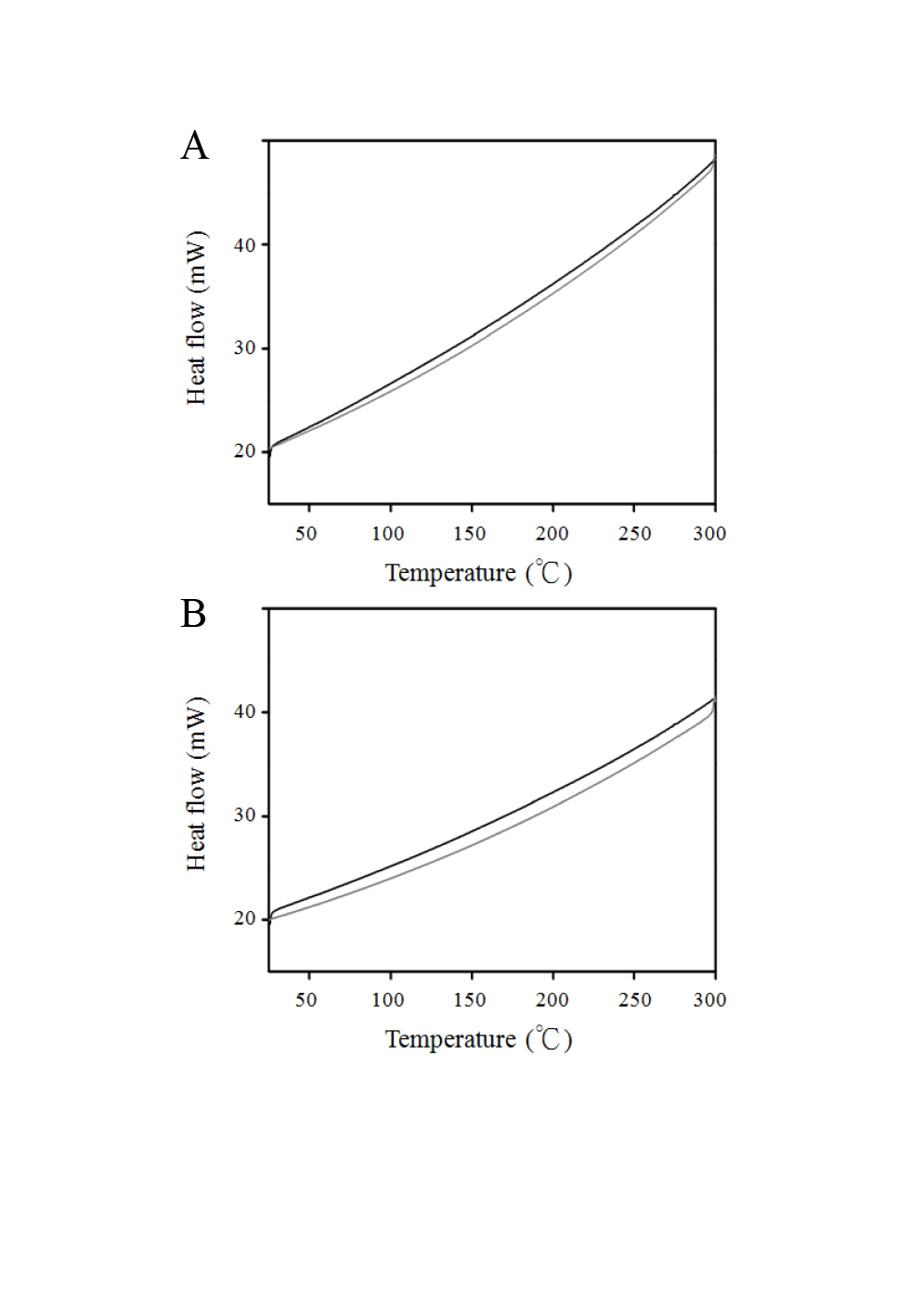

Supplement: Figure S2 — DSC thermographs of firefly light organ and the excreted uric acid of gecko. A) The light organ of L. cerata and B) the excreted uric acid of H. stejnegeri are examined under a heating (black line) and cooling (gray line) process. (TIF) [file pone.0056406.s002.tif]
